# Age at menarche and current substance use among Canadian adolescent girls: results of a cross-sectional study

**DOI:** 10.1186/1471-2458-12-195

**Published:** 2012-03-16

**Authors:** Ban Al-Sahab, Chris I Ardern, Mazen J Hamadeh, Hala Tamim

**Affiliations:** 1 Kinesiology & Health Science, York University, Ontario, Canada

## Abstract

**Background:**

Substance use is among the key public health threats that find its genesis during adolescence. Timing of puberty has been lately researched as a potential predictor of subsequent substance abuse. The present study, therefore, aims to assess the effect of age at menarche on current practices of smoking, alcohol drinking and drug use among 14-15 year old Canadian girls.

**Methods:**

The analysis of the study was based on all female respondents aged 14 to 15 years during Cycle 4 (2000/2001) of the National Longitudinal Survey of Children & Youth (NLSCY). The main independent variable was age at menarche assessed as the month and year of the occurrence of the first menstrual cycle. The dependent variables were current smoking, heavy alcohol drinking in the past 12 months and drug use in the past 12 months. Three logistic regression models were performed to investigate the association between age at menarche and each of the substance use outcomes, adjusting for possible confounders. Bootstrapping was performed to account for the complex sampling design.

**Results:**

The total weighted sample included in the analysis represented 295,042 Canadian girls. The prevalence of current smokers, heavy drinkers (drunk in the past 12 months) and drug users in the past 12 months was approximately 22%, 38% and 26%, respectively. After adjusting of all potential confounders, no association was found between age at menarche and any of the substance use outcomes. School performance and relationship with the father, however, stood out as the main variables to be associated with smoking, heavy drinking and drug use.

**Conclusions:**

Qualitative studies understanding the social and psychological changes experienced by early maturing Canadian adolescents are warranted to identify other correlates or pathways to substance use in this higher risk population.

## Background

Adolescence is a critical time period characterized by physiological changes, cognitive development and psychological transition that shapes the personality, behaviours and future health outcomes of youth [1-3]. It can also be considered as a high risk period whereby adolescents initiate and engage in different activities and behaviours that affect their life trajectories [2,4,5]. Substance use, as an example, is among the key public health threats that find its genesis in adolescence [3]. It has been suggested that 80% of smokers have initiated their smoking habit in adolescence [4], while 80% to 90% of the western population have started alcohol drinking by the age of 18 years [6].

According to the World Health Organization (WHO), substance use is globally on the rise especially among adolescents. WHO estimates that one third of the world's population aged 15 and above are smokers, 76.3 million have alcohol use disorders, and 15.3 million have drug use disorders [7]. Substance use, especially when initiated at a young age, is a risk factor for human health and wellbeing [8]. It has been associated with intentional and unintentional injuries [9], suicide [3] and mental health problems [3]. Smoking, more specifically, is a known risk factor for cardiovascular disease and certain types of cancers [10]. Alcohol drinking, on the other hand, has been linked to unsafe sex [11], teen pregnancies [11], liver cancer and epilepsy [7]. Moreover, injecting illicit drugs increases the likelihood of acquiring infectious diseases like Human Immuno-deficiency Virus (HIV) and hepatitis [3].

Given the global rise and health implications of substance use, researchers have extensively looked at its determinants with the purpose of identifying high risk populations for effective interventional programs. Particular focus has been given to the adolescence period as it predicts substance abuse in adulthood [5]. Since puberty is a critical transitional phase in the life of adolescents, timing of puberty, especially among girls, has been lately researched as a potential predictor of subsequent substance abuse [2,4]. Study results reveal that early maturing girls are at a higher risk of initiating substance use in mid-adolescence [5,12]. In a study of 496 American girls followed up from 11 years of age until 15 years revealed that girls with early menarche, a proxy measure for puberty, were 1.84 times (p-value < 0.0001) more likely to suffer from alcohol abuse than late maturers. Another longitudinal study reported that early maturing girls were 40% more likely to initiate smoking and/or drinking by the age of 13 years [5]. The association between early maturation and substance use has been explained by hormonal effects on the cognition and behaviour of the adolescents [4,13], stress due to social and psychological immaturity [14,15] and affiliation with older peers [4,5,12].

Despite the literature on the impact of puberty timing on substance use, most studies were based on bivariate associations [1,2,4,10,12,16,17]. Moreover, since social stress is associated with early menarche [5,9,13] as well as with substance use [18-20], it is important to understand this association in different social contexts [21]. To our knowledge, this is the first study to assess the relationship within the Canadian population, as all previous studies were conducted either in the United States [1,2,4,8,9,14,21-23], Australia [9], or Europe [5,10,16,17,24]. Therefore, the primary aim of this analysis is to assess the effect of age at menarche on current practices of smoking, alcohol drinking and drug use among 14-15 year old Canadian girls.

## Methods

The present study was based on the "National Longitudinal Survey of Children and Youth" (NLSCY). The NLSCY study was initiated in 1994 by Statistics Canada and Human Resources and Social Development Canada [25]. It aimed at assessing the development, health and wellbeing of Canadian children and youth. The study started with a representative sample of children aged 0 to 11 years from the 10 provinces of Canada. Since 1994 and to date, the children are being followed up through cycles of two-year intervals. At all cycles, the NLSCY administers different questionnaires for the person most knowledgeable (PMK) of the index child and/or the index child himself/herself. The NLSCY has been approved by the Chief Statistician of Statistics Canada, after going through various stages of internal reviews by advisory experts and field testing. Further information on NLSCY is available in the NLSCY user guide [25].

The study participants were female respondents aged between 14 and 15 years of age at cycle 4 (2000/2001). The main independent variable was age at menarche, self-reported by the girls in cycle 4 as the month and year of the occurrence of the first menstrual cycle. It was collected in the "self-complete questionnaires" which the child completes alone and places in a sealed envelope to ensure confidentiality. Age at menarche was categorized into three groups: early, average and late menarche. The age limit for early and late maturation was determined as approximately 1 standard deviation (SD) away from the mean age at menarche [26,27]. Premenarcheal girls were classified in the late menarcheal group [26].

Substance use has been assessed in the NLSCY using different measures. For the purpose of this study, current practices of smoking, heavy alcohol drinking and drug use were considered as the main dependent variables. The three outcomes were used as dichotomous variables (yes or no). Current smoking was assessed through the question "Which of the following describes your experience with smoking cigarettes" and the response categories were i) never smoked, ii) only had a few puffs, iii) I don't smoke anymore, iv) a few times a year, v) about once or twice a month, vi) about 1-2 days a week, vii) about 3-5 days a week, and viii) about 6-7 days a week. The first three categories were grouped to represent non-smokers and the rest as current smokers. Heavy alcohol drinking was defined, on the other hand, through the question "during the past 12 months, how often have you been drunk?". Respondents who answered "never" to the question were classified as non-heavy drinkers, whereas those who had been drunk once or more were classified as heavy drinkers. Drug use was defined through a series of questions inquiring about intake of: i) marijuana and cannabis products (also known as a joint, pot, grass or hash), ii) hallucinogens like LSD/acid and magic mushroom, iii) glue or solvents, iv) drugs without a prescription or advice from a doctor (downers, uppers, tranquilizers, ritalin, etc), and v) other drugs like crack, cocaine, heroin, speed or ecstasy, etc. Girls taking any of the above drugs at least once in the past 12 months were considered to be current drug users. All of the substance use questions were reported by the index child in the "self-complete questionnaires".

A list of covariates that have been found to be associated with substance use were assessed in this study. These variables include socioeconomic factors such as household income, PMK education, and residence (rural vs. urban). Household income was categorized by Statistics Canada into 5 levels (lowest, lower middle, middle, upper middle, and highest) based on the household size and the total household income from all sources in the past 12 months. Demographic factors, specifically, province of residence, PMK immigration status, and family type were also examined in the analysis. Family type was defined as whether the child was living with both of his/her parents (biological, step or foster parents). All of the socioeconomic and demographic variables were reported by the PMK. Finally, factors relating to the child including work for pay, relationship with the parents, school performance, and general self score were adjusted as potential confounders. General self score was imputed by Statistic Canada based on four questions adopted from the General Self-image Scale of the Marsh Self-description Questionnaire developed by H.W Marsh [28]. All of the child related factors, in exception to school performance which was collected from the PMK, was reported by the index child in the "self-complete questionnaires".

Kaplan Meier analysis employing population weights was used to estimate the mean, median and standard deviation (SD) of age at menarche. Accordingly, the distribution of early, average and late menarche was calculated. The prevalence of current and ever use of the different substance use outcomes was also assessed using population weights. Population weights estimate the number of people not selected in the sample that have been represented by each person in the sample. It also takes into consideration non-response in the survey [25]. Differences in the distribution of smoking, heavy alcohol drinking and drug use across the different levels of age at menarche and other covariates were examined using chi-square tests by applying normalized weights. Three logistic regression models were used to investigate the association between age at menarche and each of the three outcomes, adjusting for all the above-mentioned covariates. Adjusted odds ratios and 95% confidence intervals were reported for the final models. To account for the complex sampling design, bootstrapping was performed. Population weights, normalized weights, and bootstrap weights were all based on the cross-sectional weights at cycle 4 (2000-2001) that were created by Statistics Canada and provided with the NLSCY data file. All analyses with the exception of bootstrapping were conducted using the Statistical Package for the Social Sciences, version 18.0 (SPSS Inc., Chicago, Illinois). Bootstrapping was performed using the Statistical Analysis System, version 9.2 (SAS Institute, Inc., Cary, North Carolina). Statistical significance for all analyses was set at *α *< 0.05.

## Results

A total of 922 girls aged 14-15 years, weighted to represent 394,712 Canadian girls, responded to the cross-sectional survey of cycle 4. Complete information on age at menarche, however, was available among 696 (75.5%) of these girls. The vast majority of the 14-15 year olds (94.7%, 95% CI: 91.50-97.68) had started menstruating at the time of cycle 4. Using Kaplan Meier analysis, the estimated mean and median of age at menarche was 12.73 years (SD = 1.19) and 12.75 years, respectively. The proportions of early (< 11.54 years) and late maturers (> 13.92 years) were 13.4% (95% CI: 9.58-17.12) and 14.6% (95% CI: 10.44-18.87), respectively. Girls who had missing information on any of the three main substance use outcomes were further excluded from the sample. A total of 671 girls (72.7%) representing 295,042 Canadian girls aged 14-15 years were included in the analysis.

As illustrated in Table 1 almost half of the girls have ever had a cigarette and 22.0% of them are current smokers. The majority (77.4%) of the 14-15 year olds have had alcohol, resulting in a prevalence of heavy drinking of 37.7% within the study sample. A quarter of the girls were also current drug users. Table 2 represents the unadjusted association between age at menarche and the three substance use outcomes along with the other potential confounders. The distribution of smoking, heavy drinking and drug use was 30.9%, 46.2% and 30.0%, respectively, among early maturing girls. As for girls in the late menarche group, the proportion of smokers, heavy drinkers and drug users were 15.6%, 31.6%, and 11.0%, respectively. Although substance use increased with a decrease in age at menarche, no statistical association was found between age at menarche and any of the three substance use outcomes. Moreover, almost none of the demographic and socioeconomic covariates were significantly associated with smoking, heavy drinking and drug use. Factors related to the child were the only variables that proved significant at the bivariate level. After adjusting for all covariates, age at menarche remained insignificantly associated with substance use outcomes (Table 3). Only school performance and relationship with the father were found to explain variation in the dependent variables. Girls who were not very close to their fathers were more than three times as likely to be heavy drinkers and drug users (OR = 3.29, 95% CI: 1.32-8.21 and OR = 3.82, 95% CI: 1.31-11.51, respectively). Finally, having an average or lower performance in school was also associated with a higher likelihood of smoking (OR = 3.25, 95% CI: 1.58-6.70), heavy alcohol drinking (OR = 2.08, 95% CI: 1.09-3.97) and drug use (OR = 3.02, 95% CI: 1.46-6.22).

**Table 1:** Distribution of ever and current practices of smoking, alcohol drinking and drugs among 14-15 year-old Canadian girls

	N*	% (95% CI)†
**Smoking**		
Ever had a cigarette	148,477	50.3 (44.6-56.1)
Current smoking	64801	22.0 (17.6-26.3)
**Alcohol drinking**		
Ever had alcohol	228,460	77.4 (72.0-82.8)
Current drinking status	127,633	43.3 (37.4-49.1)
Current heavy drinking	111,371	37.7 (32.3-43.2)
**Drug use**		
Ever used drugs	92,131	31.2 (26.3-36.1)
Current drug use	76,192	25.8 (21.1-30.6)
Current use of cannabis	70,510	23.9 (19.3-28.5)
Current use of hallucinogens	20,291	6.9 (4.5-9.3)
Current use of glue or solvents	6,016	2.0 (0.6-3.5)
Current use of drugs without a prescription	13,581	4.6 (2.7-6.5)
Current use of other drugs	15,291	5.2 (2.9-7.5)

* Sample size is estimated using population weights

† 95% CI were calculated using bootstrapping technique

**Table 2:** Bivariate association between substance use outcomes, age at menarche and other potential covariates among 14-15 year old Canadian girls

	Current Smoking		Drunk in the past 12 months		Drugs in the past 12 months	
	N*(%)**	**OR (95% CI)**†	N*(%)**	**OR (95% CI)**†	N*(%)**	**OR (95% CI)**†
Age at menarche						
Early	28 (30.9)	2.42 (0.76-7.64)	41 (46.2)	1.86 (0.74-4.68)	27 (30.0)	3.46 (0.85-14.07)
Average	104 (21.6)	1.48 (0.55-4.00)	181 (37.4)	1.30 (0.62-2.72)	136 (28.1)	3.15 (0.84-11.83)
Late	15 (15.6)	1	31 (31.6)	1	11 (11.02)	1
Household income						
Lowest & Lower middle	10 (21.6)	1.44 (0.47-4.43)	21 (45.0)	1.68 (0.50-5.66)	10 (22.5)	0.88 (0.26-3.00)
Middle	41 (25.6)	1.80 (0.84-3.89)	69 (43.2)	1.56 (0.81-3.01)	42 (26.4)	1.09 (0.53-2.27)
Upper middle	58 (25.7)	1.81 (0.94-3.49)	85 (37.8)	1.24 (0.69-2.23)	61 (27.2)	1.14 (0.62-2.07)
Highest	39 (16.1)	1	79 (32.8)	1	60 (24.8)	1
Residence						
Rural	19 (24.9)	1.20 (0.67-2.17)	38 (48.6)	1.66 (0.95-2.89)	27 (35.6)	1.69 (0.95-3.03)
Urban	128 (21.6)	1	216 (36.3)	1	146 (24.6)	1
PMK education						
Less than secondary	20 (28.9)	2.01 (0.84-4.86)	28 (41.0)	1.51 (0.69-3.28)	20 (29.2)	1.56 (0.65-3.72)
Secondary school graduation	38 (24.4)	1.60 (0.80-3.18)	65 (41.8)	1.56 (0.86-2.81)	40 (25.7)	1.30 (0.69-2.47)
Beyond high school	39 (24.8)	1.64 (0.83-3.24)	62 (39.3)	1.40 (0.75-2.62)	52 (32.9)	1.85 (0.94-3.62)
College or university degree	47 (16.8)	1	90 (31.6)	1	59 (20.9)	1
Province of residence‡						
Eastern	16 (28.8)	**2.07 (1.06-4.06)**	21 (38.2)	0.83 (0.45-1.54)	15 (27.4)	1.35 (0.66-2.77)
Central	100 (23.6)	1.58 (0.90-2.79)	151 (35.5)	0.74 (0.43-1.26)	116 (27.4)	1.35 (0.75-2.43)
Western	31 (16.3)	1	82 (42.7)	1	42 (21.8)	1
PMK immigration status						
No	131 (23.8)	2.11 (0.83-5.40)	215 (39.0)	1.67 (0.75-3.73)	149 (27.1)	1.61 (0.69-3.74)
Yes	14 (12.9)	1	31 (27.7)	1	21 (18.7)	1
Living with both parents						
No	38 (29.5)	1.66 (0.91-3.04)	59 (45.6)	1.50 (0.81-2.76)	43 (33.1)	1.56 (0.82-2.99)
Yes	109 (20.1)	1	194 (35.9)	1	130 (24.1)	1
Relationship with mother						
Very close	52 (16.0)	1	102 (31.2)	1	66 (20.3)	1
Somewhat close	71 (25.4)	**1.79 (1.02-3.13)**	116 (41.6)	1.57 (0.97-2.53)	86 (30.7)	**1.74 (1.01-3.00)**
Not very close	21 (37.4)	**3.12 (1.10-8.89)**	31 (54.4)	2.63 (0.91-7.57)	20 (34.2)	2.04 (0.76-5.44)
Relationship with father						
Very close	32 (14.1)	1	65 (28.6)	1	36 (15.9)	1
Somewhat close	60 (20.8)	1.61 (0.87-2.97)	108 (37.6)	1.50 (0.86-2.62)	77 (26.8)	**1.94 (1.09-3.44)**
Not very close	42 (39.5)	**3.99 (1.95-8.18)**	62 (57.6)	**3.39 (1.68-6.85)**	45 (41.9)	**3.82 (1.88-7.76)**
Work for pay						
No	32 (25.0)	1	42 (33.1)	1	28 (22.1)	1
Yes	116 (21.6)	0.83 (0.44-1.56)	212 (39.4)	1.32 (0.79-2.20)	146 (27.1)	1.31 (0.72-2.38)
School performance						
Very well/Well	58 (13.9)	1	125 (29.9)	1	78 (18.6)	1
Average/Poor/Very poor	80 (35.0)	**3.33 (1.96-5.68)**	118 (51.3)	**2.47 (1.49-4.10)**	84 (36.9)	**2.57 (1.56-4.23)**
Self score	**-**	**0.86 (0.78-0.95)**	**-**	**0.89 (0.82-0.96)**	**-**	**0.89 (0.81-0.97)**

* Sample size is estimated using normalized weights

** Frequencies are row percentages estimated using normalized weights.

† 95% CI were calculated using bootstrapping technique

‡ Eastern Atlantic: Newfoundland & Labrador, Nova Scotia, Prince Edward Island & New Brunswick; Eastern Central: Quebec & Ontario; Western Prairies: Manitoba, Saskatchewan, & Alberta; Western British Columbia: British Columbia

**Table 3:** Logistic regression for substance use outcomes and age at menarche among 14-15 year old Canadian girls

	Current Smoking	Drunk in the past 12 months	Drugs in the past 12 months
	**OR (95% CI)**†	**OR (95% CI)**†	**OR (95% CI)**†
Age at menarche			
Early	2.49 (0.64-9.74)	1.77 (0.49-6.38)	5.97 (0.36-99.16)
Average	1.65 (0.57-4.78)	1.13 (0.44-2.88)	5.40 (0.37-79.01)
Late	1	1	1
Household income			
Lowest & Lower middle	0.58 (0.09-3.83)	0.15 (0.02-1.17)	0.10 (0.01-4.80)
Middle	1.13 (0.41-3.10)	0.82 (0.32-2.10)	0.57 (0.20-1.62)
Upper middle	1.38 (0.59-3.22)	0.86 (0.39-1.86)	0.73 (0.33-1.62)
Highest	1	1	1
Residence			
Rural	0.89 (0.40-1.97)	1.58 (0.75-3.33)	1.63 (0.71-3.73)
Urban	1	1	1
PMK education			
Less than secondary	1.57 (0.49-5.00)	0.98 (0.32-3.02)	1.30 (0.36-4.70)
Secondary school graduation	1.15 (0.49-2.68)	1.25 (0.58-2.69)	1.14 (0.53-2.45)
Beyond high school	0.98 (0.34-2.81)	1.23 (0.55-2.78)	1.61 (0.66-3.88)
College or university degree	1	1	1
Province of residence‡			
Eastern	2.51 (0.93-6.77)	0.84 (0.38-1.84)	2.20 (0.86-5.59)
Central	1.73 (0.76-3.94)	0.78 (0.40-1.50)	2.09 (0.98-4.50)
Western	1	1	1
PMK immigration status			
No	2.15 (0.61-7.53)	1.64 (0.51-5.22)	1.98 (0.67-5.87)
Yes	1	1	1
Living with both parents			
No	1.40 (0.58-3.41)	2.23 (0.83-5.99)	2.26 (0.80-6.40)
Yes	1	1	1
Relationship with mother			
Very close	1	1	1
Somewhat close	1.27 (0.56-2.88)	0.93 (0.48-1.78)	1.10 (0.52-2.33)
Not very close	0.89 (0.21-3.73)	1.38 (0.26-7.25)	0.69 (0.13-3.75)
Relationship with father			
Very close	1	1	1
Somewhat close	1.25 (0.51-3.06)	1.81 (0.89-3.70)	1.92 (0.83-4.46)
Not very close	2.67 (0.99-7.20)	**3.29 (1.32-8.21)**	**3.82 (1.31-11.15)**
Work for pay			
No	1	1	1
Yes	1.41 (0.60-3.28)	1.74 (0.87-3.51)	1.92 (0.83-4.47)
School performance			
Very well/Well	1	1	1
Average/Poor/Very poor	**3.25 (1.58-6.70)**	**2.08 (1.09-3.97)**	**3.02 (1.46-6.22)**
Self score	0.95 (0.83-1.09)	0.95 (0.85-1.07)	0.98 (0.86-1.11)

† 95% CI were calculated using bootstrapping technique

‡ Eastern Atlantic: Newfoundland & Labrador, Nova Scotia, Prince Edward Island & New Brunswick; Eastern Central: Quebec & Ontario; Western Prairies: Manitoba, Saskatchewan & Alberta; Western British Columbia: British Columbia

## Discussion

The present paper examined the relationship between age at menarche and current practices of smoking, heavy drinking and drug use among 14-15 year old females in Canada. The prevalence of smokers, heavy drinkers and drug users in the study sample was approximately 22%, 38% and 26%, respectively. After adjusting of all potential confounders, no association was found between age at menarche and any of the substance use outcomes. School performance and relationship with the father, however, stood out as the main variables to be associated with smoking, heavy drinking and drug use.

The prevalence of substance use among 14-15 year old Canadian girls reported in this study is higher than previous Canadian estimates. Using the NLSCY data (cycle 3, 1998/1999), Hotton & Haans (2004) reported alcohol intoxication in the past 12 months among girls (12-15 years) was 19.0% as compared to 37.7% in the present study [29]. In the same study, the prevalence of drug use in the past 12 months was half (18.3%) of the rate reported in this study. As for smoking, the results of the Canadian Tobacco Use Monitoring Survey (2003) suggest that 20% of the females aged 15-19 years were smokers [30].

Despite the high prevalence of substance use outcomes, age at menarche in the present study did not explain any variation in the current practices of smoking, heavy drinking and drug use. Previous literature, in contrast, has revealed that early pubertal timing increases the risk of substance use. A longitudinal study from the United States, revealed that early developing girls in the 7^th ^grade were 2.7 times (95% CI: 1.6-4.4) more likely to have been drunk in the past 12 months, 2.5 times (95% CI: 1.5-4.3) more likely to have ever tried marijuana and 1.6 times (95% CI: 1.3-2.0) more likely to have ever tried cigarettes [2]. A more comprehensive cross-sectional study by Patton et al. (2004) assessed the effect of pubertal development based on tanner stages (I to V) on substance use among 10-15 year old boys and girls in the United States and Australia [9]. Based on the findings of the study, lifetime substance use was almost twice as high (OR = 1.7, 95% CI: 1.42-2.1) in adolescents undergoing mid-tanner stages (III) and three times (OR = 3.1, 95% CI: 2.4-4.2) as likely among those undergoing more advanced tanner stages (IV/V) as compared to their counterparts undergoing I/II tanner stages. Recent substance use, however, was less pronounced in adolescents going through stage III (OR = 1.4, 95% CI: 1.0-1.9) but more strongly associated with those in their stage IV and V (OR = 2.3, 95% CI: 1.7-3.3) [9].

The association between early puberty and substance use has been attributed to biological, psychological and social factors. It has been hypothesized that pubertal hormones have effects on the cognition and behaviour of the adolescents which consequently contribute to substance use [4,13]. Early maturing girls might also engage in substance use because of the pressure of the misbalance between the adult appearance and the social and psychological immaturity [14,15]. Girls who perceive their tempo of puberty as unusual from their peers might experience high levels of stress which might lead to inappropriate decisions, like substance use [4]. Moreover, girls who mature earlier are thought to affiliate with older peers and hence imitate their behaviour [4,5,12].

Interestingly, in spite of the evidence of the previous literature and supporting hypotheses of the association between puberty and substance use, no relationship was found in the present study. The explanation for this finding is unclear without further quantitative studies to confirm these results. Qualitative studies on social and psychological changes experienced by early maturing Canadian adolescents are also warranted to identify other correlates or pathways to substance use in this higher risk population. Nevertheless, examining longitudinally the substance use trajectories among the different menarche groups adds further insight to the subsequent impact of age at menarche on adult substance use. A plausible explanation maybe that the emotional stress associated with early maturation might not have an effect on substance use. This is consistent with a Canadian study by Hotton and Haans (2004) that suggests that emotional problems do not influence adolescent substance use [29]. In this study, the authors found that youth with emotional problems were less likely to be drunk in the past year than their counterparts [29]. Further to the above, self-esteem which is associated with early puberty [5] was not found in the present study to be significantly associated with any of the substance use outcomes. Moreover, it has been hypothesized that as girls grow older the association between age at menarche and substance use practices dissipates [12]. Since the present study assesses this association during mid-adolescence, the disagreement of the results with the literature might be due to the assessment of this association with substance use initiation at younger ages. Further to the above, this disagreement might be attributed to the multivariate analysis conducted in this study. As mentioned earlier, most studies examining the relationship between substance use outcomes and puberty were based on bivariate associations [1,2,4,10,12,16,17].

In this study, school performance and relationship with the father, rather than age at menarche, played an important role in explaining practices of substance use. The results are consistent with another Canadian study that was conducted using the NLSCY, cycle 3 data [29]. Students having poor or very poor grades at school were 2.35 times (95% CI: 1.21-4.54) more likely to have drunk to intoxication than those having good or very good grades at school. Other studies have also shown an association between low school commitment and substance use [31,32]. The Canadian study has also shown that hostile and negative parenting styles were associated with drug use (OR = 1.09, 95% CI: 1.02-2.16) and alcohol intoxication (OR = 1.11, 95% CI: 1.03, 1.19) [29]. A review paper investigating the relationship between adolescent alcohol consumption and parenting strategies, revealed that good parent-child relationship delays alcohol initiation (p < 0.002) and decreases future levels of alcohol use (p < 0.001) [33].

The response rate at cycle 4 was 85.4%. However, the cross-sectional weights used in the analysis takes into account for the non-response. In the present study, girls for whom information on age at menarche and substance use outcomes was missing were excluded from the analysis. Therefore, the percentage of girls with complete data was 72.7%. Girls excluded from the analysis were compared in terms of socioeconomic, demographic, and child-related factors to those girls included in the analysis. No statistical difference, except for province, was detected between the two groups (data not shown). Girls with missing data were more likely to be residents of the Central provinces. Another limitation of the study is the cross-sectional design of the study where the temporal relationship between the outcomes and the covariates cannot be determined. The outcome variables, also, were measured in a limited time frame (within the past 12 months). Future studies examining longitudinally the substance use trajectories focusing on the frequency of substance use are warranted. Further to that, the substance use outcomes were dichotomous in nature. Since the three outcomes were assessed in the survey using different measures, a two-level variable allowed the comparability of the substance use outcomes. Some potential confounders were also missing from the analysis. Peer substance use, an important determinant of substance use, was collected in the NLSCY but not included in the analysis due to its high co-linearity with the outcome variables. Parental substance use was not available. Only PMK's smoking and alcohol drinking practices were collected in the NLSCY. Moreover, there was no access to academic records from schools and school performance had to be collected by the PMK. Finally, the study results are reflective of the years 2000/2001. Although more recent cycles of the NSCLY are available for the year 2006/2007, generalizability of such results would be limited to the original NLSCY cohort that was sampled in 1994. At each cycle, Statistics Canada creates cross-sectional weights to account for demographic changes in the population. These weights allow the generalizability of the results to the Canadian population. No cross-sectional weights, however, were created after cycle 4. Nevertheless, this is the first study that looks at the association between age at menarche and substance use in the Canadian population. The sample size of the study, the multivariate analysis and the decreased likelihood of recall bias stand out as the main strengths. Age at menarche was reported at an age (14-15 years) close to the menarche event. Based on Koo (1997), the accuracy of reporting age at menarche increases with a decrease in recall interval [34].

## Conclusions

The present study revealed no association between age at menarche and practices of smoking, heavy alcohol drinking and drug use among Canadian adolescent girls. School performance and relationship with the father played an important role in explaining variation in the substance use outcomes. School interventions, therefore, should aim to improve school experiences to reduce the prevalence of substance use among the youth. The schools should also aim to involve parents in these interventions since parental relationships have been shown to be effective in limiting substance use practices. Moreover, qualitative studies understanding the social and psychological changes experienced by early maturing Canadian adolescents are warranted to identify other correlates or pathways to substance use in this higher risk population.

## Abbreviations

CI: Confidence interval; HIV: Human Immunodeficiency Virus; NLSCY: National Longitudinal Survey of Children & Youth; OR: Odds ratio; PMK: Person most knowledgeable; SAS: Statistical Analysis Software; SD: Standard deviation; SPSS: Statistical Package for Social Sciences.

## Competing interests

The authors declare that they have no competing interests.

## Authors' contributions

BAS: Generated the idea and the design of the study; performed analysis and write up of the paper. CIA: Made substantial contributions to the design and interpretation of the results; provided technical support and advice; reviewed the final paper and all its drafts. MJH: Made substantial contributions to the design and interpretation of the results; provided technical support and advice; reviewed the final paper and all its drafts. HT: Generating the idea and design of the study; supervised the analysis, interpretation of the results and write up of the manuscript; reviewed the final paper and all its drafts.

All authors read and approved the final manuscript.

## Pre-publication history

The pre-publication history for this paper can be accessed here:

http://www.biomedcentral.com/1471-2458/12/195/prepub
